# Ohio Medical College

**Published:** 1849-07

**Authors:** 


### OHIO MEDICAL COLLEGE.

It was announced, a short time since, that the chairs in the Ohio Medical College had all been declared by the Board of Trustees vacant, preparatory to a reorganization of the Faculty. We learn from the public prints that the former professors have been restored, and that Dr. Drake and Dr. George W. Bayless have been added to their number. Dr. Drake takes the chair of Theory and Practice, and Dr. Bayless has been appointed to the chair of Descriptive Anatomy. We congratulate the friends of the school on the accession of so much talent to its able corps of teachers. Dr. Drake is now entirely at home—in the city and the chair of his choice, and in the school which he founded. He has our fervent wishes for long life and unvarying prosperity. Dr. Bayless is a practiced and thorough anatomist, and a good lecturer, and with his industry and ambition to excel will not fail in the duties of his chair. The Ohio Medical College, one of the oldest institutions in the West, is so identified with our profession that no one who takes an interest in the latter can feel indifferent to the fame of the former. Every friend of true medical science must wish the college a speedy and complete triumph over those malign influences which have hitherto hindered its progress.

